# Ridge Tillage Improves Soil Properties, Sustains Diazotrophic Communities, and Enhances Extensively Cooperative Interactions Among Diazotrophs in a Clay Loam Soil

**DOI:** 10.3389/fmicb.2020.01333

**Published:** 2020-06-30

**Authors:** Xiaojing Hu, Aizhen Liang, Qin Yao, Zhuxiu Liu, Zhenhua Yu, Guanghua Wang, Junjie Liu

**Affiliations:** ^1^Key Laboratory of Mollisols Agroecology, Northeast Institute of Geography and Agroecology, Chinese Academy of Sciences, Harbin, China; ^2^University of Chinese Academy of Sciences, Beijing, China

**Keywords:** no tillage, ridge tillage, moldboard plow tillage, diazotrophic communities, diazotrophs’ network analysis

## Abstract

Reduced tillage practices [such as ridge tillage (RT)] have been potential solutions to the weed pressures of long-term no tillage (NT) and the soil-intensive disturbances caused by conventional tillage [such as moldboard plow (MP) tillage]. Although soil diazotrophs are significantly important in global nitrogen (N) cycling and contribute to the pool of plant-available N in agroecosystems, little is currently known about the responses of diazotrophic communities to different long-term tillage practices. In the current study, we investigated the differences among the effects of NT, RT, and MP on soil properties, diazotrophic communities, and co-occurrence network patterns in bulk and rhizosphere soils under soybean grown in clay loam soil of Northeast China. The results showed that RT and MP led to higher contents of total C, N, and available K compared to NT in both bulk and rhizosphere soils, and RT resulted in higher soybean yield than NT and MP. Compared to NT and RT, MP decreased the relative abundances of free-living diazotrophs, while it promoted the growth of copiotrophic diazotrophs. Little differences of diazotrophic community diversity, composition, and community structure were detected between RT and NT, but MP obviously decreased diazotrophic diversity and changed the diazotrophic communities in contrast to NT and RT in bulk soils. Soil nitrogenous nutrients had negative correlations with diazotrophic diversity and significantly influenced the diazotrophic community structure. Across all diazotrophs’ networks, the major diazotrophic interactions transformed into a cooperatively dominated network under RT, with more intense and efficient interactions among species than NT and MP. Overall, our study suggested that RT, with minor soil disturbances, could stabilize diazotrophic diversity and communities as NT and possessed highly positive interactions among diazotrophic species relative to NT and MP.

## Introduction

Tillage practices, i.e., conventional tillage and conservational tillage, are defined by the degree of soil inversion and the amount of crop residues remaining on the ground for the purposes of crop production and sustainable agricultural environments (Sengupta and Dick, 2015; Zuber and Villamil, 2016). Conventional tillage using moldboard plow (MP) involves mechanical soil inversion that mixes plant residues into the tillage layer and controls weed growth (Hobbs et al., 2008); however, long-term intensive tillage can accelerate the decomposition of organic materials and cause severe degradation of the soil structure (Liu et al., 2010). In contrast, the application of conservational tillage, including no tillage (NT), is an alternative strategy to curb or reverse soil degradation, minimize soil erosion risks, decrease soil disturbance, enhance the soil regulatory capacity, and reduce energy or fuel costs (Busari et al., 2015). Despite the various benefits of NT, concerns have arisen about impeding the proper development of crop roots, outbreaks of herbicide-resistant weed populations, and increasing incidence of stubble-borne disease (Thomas et al., 2007; Martínez et al., 2008). Thus, a variety of “reduced tillage” practices [such as ridge tillage (RT)] that served as conservational tillage can maximize the positive effects on soil quality to improve and sustain productivity and increase food security and profits (Kuntz et al., 2013). These reduced-pass practices can break the compacted soil surface associated with NT, avoid the intense soil perturbations that occur under moldboard tillage, and largely maintain soil health and increase soil resiliency compared with other tillage practices (Busari and Salako, 2015).

Tillage practices affect the soil physicochemical properties and therefore influence the habitat of soil microorganisms in various ways. Thus, the changes in microbial communities can, in turn, reflect soil fertility and nutrient cycling through achieving many important ecological functions (Jiang et al., 2011; Fierer, 2017). Conventional tillage periodically inverts and redistributes the soil nutrients uniformly throughout the tilled layer (Chivenge et al., 2007; Sun H. et al., 2016). The homogenized soils likely lead to decreases in microbial biomass and diversity (Balota et al., 2003; Helgason et al., 2009; Sengupta and Dick, 2015), especially the disruption of fungal mycelia by intensive tillage disturbances (Cookson et al., 2008). Despite these, microbes may be possibly more accessible to crop residues due to conventional tillage, thus inducing the increase in microbial activity (Zuber and Villamil, 2016). Conventional tillage is linked to a selection of aerobic microorganisms and fast-growing copiotrophs (Mathew et al., 2012; Carbonetto et al., 2014). In contrast, conservational tillage creates pronouncedly different habitats for various microorganisms, and the microbial population and diversity are expected to increase with the reduction in tillage (Adl et al., 2006; Helgason et al., 2009; Säle et al., 2015). In addition, mounting evidence has revealed that conservational tillage practices induce the shifts from a bacteria-dominated community to a fungi-dominated one because of the less frequent disturbance of soil compared to that under conventional tillage (Acosta-Martínez et al., 2007; Minoshima et al., 2007). Through these studies, the general changes of bacterial and fungal communities in response to different tillage practices have been proposed. However, little information is available on how and to what extent tillage practices affect soil functional microorganisms, such as soil diazotrophs.

Diazotrophs are responsible for biological dinitrogen fixation and therefore supply additional N sources to the ecosystem, which is a potential alternative to chemical N fertilizer use in agricultural systems (Wang C. et al., 2017). Detailed information on the diazotrophic community in response to long-term tillage practices would be useful for the development of their biological functions in agroecosystems. Höflich et al. (1999) found that conservational tillage could increase nodulation and dinitrogen fixation in pea plants by stimulating the activity of the *Rhizobium leguminosarum* population in sandy loam soil. Using traditional culture-dependent methods, conservational tillage had been reported to provide habitats for free-living diazotrophs and provide an especially favorable habitat for rhizospheric dinitrogen fixation (Sprent and Sprent, 1990). However, no attempt has yet been made to characterize the diazotrophic community under different tillage practices with high-throughput sequencing, which can provide deep insight into the functional diversity and species variation of microbial populations (Shendure et al., 2017). Moreover, diazotrophs do not live in isolation but instead form interspecies networks with positive or negative interactions (Chow et al., 2014). The co-occurrence networks of diazotrophs have been studied in agronomic soils, such as fertilization management and rhizosphere effects based on high-throughput sequencing technology (Fan et al., 2018; Hu et al., 2019), but still vacant in tillage practices. Therefore, a better understanding of diazotrophs’ networks is needed to reveal the interactions and co-occurrence patterns of diazotrophic species under different tillage practices.

Here we conducted a study on clay loam soil (Typic Hapludoll) under NT, RT (conservational tillage), and MP (conventional tillage) in Northeast China. Based on high-throughput sequencing of the *nifH* gene and the analysis of their corresponding diazotrophic ecological network, we aimed to address the following questions: (1) how RT and MP tillage influence the diazotrophic communities compared with NT and (2) how the network interactions among diazotrophic species changed when NT transforms to RT or MP tillage. Our main objective was to characterize and better understand the diazotrophic community and the network structure under different tillage practices, which is useful in assessing the optional tillage for achieving preferable microbial colonization and sustainable agroecosystems.

## Materials and Methods

### Experimental Design and Soil Sample Collection

The field experiment was established in 2013 at a long-term experimental station of the Northeast Institute of Geography and Agroecology, Chinese Academy of Sciences, in Changchun, Jilin Province, China (44°59′ N, 125°23′ E). The mean annual precipitation and air temperature were 614 mm and 6.4°C, respectively. Three tillage practices, NT, RT, and MP tillage, were conducted in the same location, which were arranged in a completely randomized block design with four replicates, and the area for each replicate plot was 25 × 7.8 m. Soybean is cultivated in rotation with corn in alternate years, and this cropping rotation was applied under the three tillage practices. After harvest, the soybean residues were fully and directly returned to the soil surface of the three tillage practices. The maize stalks were manually cut into approximately 30 cm in length and laid on the soil surface of each tillage practice for their corresponding crop residues. NT experienced no soil disturbance except for planting with a two-row John Deere 7200 NT planter (Moline, IL, United States). RT included ridging in June, and no other soil disturbance was conducted until the next year. In contrast to NT and RT, MP treatment involved two tillage applications after planting, with one fall moldboard plowing (approximately 20 cm in depth) after harvest and one spring disking (approximately 7.5–10 cm in depth) with ridge building before planting. For the cultivation of soybean under NT, RT, and MP, 40 kg N ha^–1^, 60 kg P ha^–1^, and 80 kg K ha^–1^ were applied as base fertilizers. For the cultivation of corn under the three tillage practices, 100 kg ha^–1^ nitrogen (N), 45.5 kg P ha^–1^, and 78 kg K ha^–1^ were applied as base fertilizers, and another 50 kg N ha^–1^ was used as top dressing at the jointing stage of corn vegetative growth. The soybean yield was determined by hand in 3-m lengths of six interior rows from each plot.

Each tillage treatment was arranged in a randomized block design with four replicate plots. Two repeated soil samples were collected from each plot, and a total of eight repeated soil samples were obtained from each tillage treatment. Specifically, nine individual soil cores (0–20 cm soil depth) were randomly collected and composited together as a repeated soil sample to minimize within-plot variation. Overall, 24 bulk and 24 rhizosphere soil samples (three tillage practices × eight replicates) were collected under soybean cultivation at the beginning pod stage of soybean reproductive growth (on 25 July 2017). Each soil sample was placed in an individual sterile plastic bag and immediately transported back to the laboratory. After passing through a 2-mm sieve, the soil sample was divided into two parts: one was stored at −80°C for DNA extraction and the other was stored at 4°C for determination of soil properties. Soil physical and chemical properties, including soil pH, total carbon (TC), total nitrogen (TN), total phosphorus (TP), total potassium (TK), available phosphorus (AP), available potassium (AK), NH_4_^+^-N, and NO_3_^–^-N, were measured as previously described in Hu et al. (2017).

### Soil DNA Extraction and Illumina MiSeq Sequencing

The total DNA was extracted from 0.5 g of each soil sample (stored at -80°C) using a FastDNA^®^ SPIN Kit for Soil (MP Biomedicals, United States) according to the manufacturer′s procedures, and the DNA concentrations were measured with a NanoDrop 2000 spectrophotometer (Thermo Scientific, United States). The extracted DNA was stored at −20°C until downstream analysis. The primers PolF (5′-TGC GAY CCS AAR GCB GAC TC-3′) and PolR (5′-ATS GCC ATC ATY TCR CCG GA-3′) were used to amplify the *nifH* gene (Poly et al., 2001). A sample-specific barcode (6 bp) was added to the forward primer to distinguish the amplified products, which were sequenced on an Illumina MiSeq PE 300 platform at Majorbio BioPharm Technology Co., Ltd., Shanghai, China. The sequences obtained from this research were deposited at NCBI with accession number SRP 226893.

The raw *nifH* nucleotide sequences were analyzed through Quantitative Insight into Microbial Ecology (QIIME) pipeline^1^ (Caporaso et al., 2010). A total of 964,583 high-quality sequences of *nifH* gene were acquired after removing the barcodes, primers, and low-quality sequences with an average quality score < 20. The obtained sequences were further translated to amino acid sequences on FunGene pipeline^2^ (Fish et al., 2013), and the amino acid sequences that did not match the *nifH* protein were discarded. The remaining sequences were clustered into operational taxonomic units (OTUs) at 95% similarity level with UPARSE (Edgar, 2013), and the taxonomy of each OTU was aligned against the *nifH* gene database^3^. A total of 10,288 OTUs were clustered from 964,583 high-quality *nifH* gene sequences (from 9,587 to 23,604) across 47 soil samples. One replicate of RT in bulk soil (RTB1) failed in the PCR production procedure, and thus it was discarded in the downstream analysis. To minimize the impact of sequence count variation among samples, a subset of 9,587 sequences per sample, based on minimum sequences, was randomly extracted prior to the downstream analysis. The relative abundance of each diazotrophic group at the phylum, genus, and species taxonomic levels was used to compare the difference and/or similarity of diazotrophic communities in response to tillage practices for the subsequent analysis.

### Network Construction and Analysis

Diazotrophic ecological networks were constructed with sequencing data through Molecular Ecological Network Analysis Pipeline^4^ based on random matrix theory methods, which were described previously (Zhou et al., 2010, 2011; Deng et al., 2012). In brief, eight replicates for each tillage practice for bulk or rhizosphere soils were uploaded into the pipeline, and only OTUs detected in more than half of the replicates were considered. In total, six diazotrophs’ networks associated with three tillage practices for bulk and rhizosphere soils were obtained with automatically generated cutoff values (similarity threshold, *S*_t_). Random networks were generated by keeping the same number of nodes and links as the above empirical networks and were used to examine the statistical significance of the network properties using the Maslov–Sneppen method (Maslov and Sneppen, 2002). The modularity of all the obtained networks was over 0.4, which suggested that they had modular structures (Newman, 2006). A node in a network represents the OTU, and an edge or link between the pair of OTUs was assigned if the correlation between their abundance exceeds the *S*_t_. The “global network properties,” “individual node centrality,” and “module separation and modularity” were calculated on the pipeline. Among these network properties, node degree or connectivity is the number of neighbors of a node, clustering coefficient represents how well a node is connected with its neighbors, density represents how densely the network is populated with edges, and a module is a group of nodes that are highly linked within the group but with few links outside the group (Zhou et al., 2011; Deng et al., 2012). In addition, the percentage of positive or negative edges in each network was calculated to examine the cooperative or the competitive interactions between diazotrophic species. Finally, based on this pipeline, we incorporated soil properties into microbial networks to measure the effects of soil properties under the three tillage practices on diazotrophs’ network structures.

### Statistical Analysis

The α-diversity of a diazotrophic community was calculated using the alpha_diversity.py function in QIIME. Significant differences in soil properties, diazotrophic taxa, and α-diversity under different tillage practices were tested by analysis of variance (ANOVA). Pearson’s correlations between the α-diversity or the abundance of diazotrophic taxa and soil properties or network complexity were determined using SPSS ver. 22.0 software. Principal coordinate analysis (PCoA) was conducted based on weighted UniFrac distances to depict the patterns of the β-diversity for diazotrophic communities in R software (ver. 3.5.1), using the “ape” package. The statistical significance of diazotrophic community structures was tested with Adonis analysis in R using “vegan” package. Mantel test was used to link the structure of a diazotrophic community with the soil properties and was conducted in R using “vegan” package. Cytoscape 3.7.2 software was used to visualize the network graphs (Shannon et al., 2003).

## Results

### Soil Properties and Soybean Yields

Except for NH_4_^+^-N, the soil properties were significantly different among NT, RT, and MP in bulk and rhizosphere soils (Table 1). Compared with NT and RT, MP significantly increased soil pH in both bulk and rhizosphere soils. Soil TC, TN, and AK were significantly higher in RT and MP than in NT of both bulk and rhizosphere soils. TP and AP were significantly higher in RT than in MP and NT of bulk soils, while they were remarkably higher in MP and RT than in NT of rhizosphere soils. In addition, MP and RT resulted in higher NO_3_^–^-N contents in bulk soils compared with that in NT, but there was no significant difference between RT and NT in rhizosphere soils.

**TABLE 1:** Effects of different tillage practices on soil properties.

**Soil properties**	**NTB**	**RTB**	**MPB**	**NTR**	**RTR**	**MPR**
pH	5.47 ± 0.01b	5.46 ± 0.03b	5.75 ± 0.04a	5.41 ± 0.01b	5.41 ± 0.01b	5.70 ± 0.03a
Total carbon (g kg^–1^)	14.1 ± 0.39b	18.3 ± 0.41a	18.7 ± 0.45a	19.2 ± 0.2c	25.5 ± 1.09b	26.5 ± 0.68a
Total nitrogen (g kg^–1^)	1.66 ± 0.09b	2.03 ± 0.11a	2.19 ± 0.23a	2.15 ± 0.05b	2.72 ± 0.06a	2.68 ± 0.07a
Carbon/nitrogen	8.54 ± 0.43a	9.06 ± 0.65a	8.58 ± 0.78a	8.95 ± 0.18c	9.38 ± 0.46b	9.90 ± 0.40a
Total phosphorus (g kg^–1^)	0.45 ± 0.00b	0.47 ± 0.01a	0.45 ± 0.02b	0.47 ± 0.02c	0.51 ± 0.01b	0.54 ± 0.01a
Total potassium (g kg^–1^)	23.4 ± 0.21a	23.3 ± 0.21ab	23.2 ± 0.22b	23.8 ± 0.29a	23.3 ± 0.24b	23.1 ± 0.23b
Available phosphorus (mg kg^–1^)	15.2 ± 1.10b	19.6 ± 0.51a	15.4 ± 0.23b	18.2 ± 1.25c	23.5 ± 0.56b	27.2 ± 1.45a
Available potassium (mg kg^–1^)	107 ± 2.6c	137 ± 1.7a	133 ± 4.0b	188 ± 1.3b	214 ± 1.7a	213 ± 1.2a
NH_4_^+^-N (mg kg^–1^)	1.71 ± 0.18a	1.75 ± 0.19a	1.78 ± 0.19a	3.45 ± 0.36a	3.47 ± 0.40a	3.17 ± 0.39a
NO_3_^–^-N (mg kg^–1^)	0.20 ± 0.06c	0.27 ± 0.05b	0.37 ± 0.07a	0.83 ± 0.12b	0.88 ± 0.16b	1.33 ± 0.22a

*The values are means of eight replicates, with different letters indicating significant differences among tillage practices for bulk or rhizosphere soil. Means were compared using ANOVA, *p* < 0.05 level. NT, no-tillage; RT, ridge tillage; MP, moldboard plow tillage; B, bulk soils; R, rhizosphere soils.*

The soybean yields were 2,406, 2,826, and 2,592 kg ha^–1^ in the NT, RT, and MP, respectively (Supplementary Figure S1). Although the average yield of RT was 17.5 and 9.03% higher than those of NT and MP, respectively, the statistical analysis showed no significant difference in soybean yield among the three tillage practices (Supplementary Figure S1).

### Diazotrophic Communities and Links to Soil Properties

**FIGURE 1:**
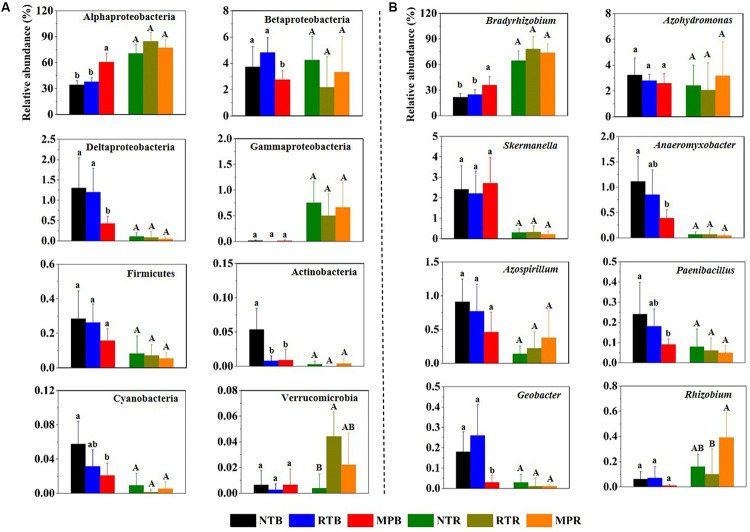
Relative abundances of main diazotrophic phyla **(A)** and genera **(B)** under different tillage practices in bulk and rhizosphere soils. NT, no-tillage; RT, ridge tillage; MP, moldboard plow; B, bulk soil; R, rhizosphere soil. The values are means of eight replicates, with different letters indicating significant differences at *p* < 0.05 (ANOVA).

Across all samples, Proteobacteria was the dominant phylum, with the relative abundances ranging from 64.6 to 91.9%, followed by high proportions of unclassified diazotrophic sequences ranging from 8.03 to 35.0%. The phyla Actinobacteria, Cyanobacteria, Firmicutes, and Verrucomicrobia occasionally occurred at low frequencies (Figure 1A). Compared with NT, MP significantly increased the relative abundance of Alphaproteobacteria but decreased the relative abundance of Betaproteobacteria, Deltaproteobacteria, Actinobacteria, and Cyanobacteria in bulk soil (Figure 1A). However, RT had little influence on these diazotrophic phyla compared to NT. Except for Verrucomicrobia, the relative abundances of these diazotrophic phyla were not significantly different among the three tillage practices in rhizosphere soil (Figure 1A). At the genus level, *Bradyrhizobium* dominated the diazotrophic community, and the changes of this genus in response to different tillage practices were consistent with its affiliated phylum (Alphaproteobacteria) in bulk and rhizosphere soils (Figure 1B). In contrast, the three tillage practices had little difference on the relative abundance of *Skermanella* and *Azospirillum* in bulk soil, which are both affiliated with Alphaproteobacteria (Figure 1B). Additionally, the correlations between soil properties and diazotrophic taxa showed that soil TC, TN, TP, AP, AK, NH_4_^+^-N, and NO_3_^–^-N significantly influenced the relative abundances of main diazotrophic phyla and genera, expect for Betaproteobacteria. Alphaproteobacteria and *Bradyrhizobium* were positively correlated with these soil properties, while other diazotrophic phyla and genera had negative correlations with these soil properties (Supplementary Figure S2).

**FIGURE 2:**
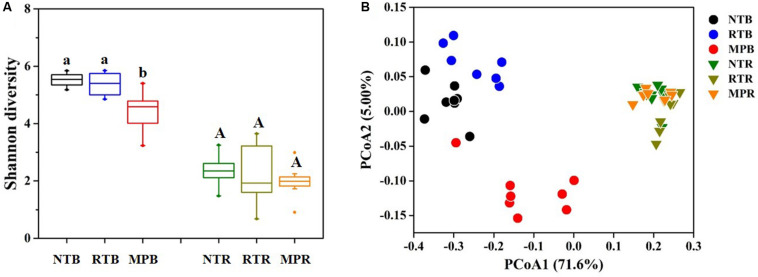
Effects of different tillage practices on α- **(A)** and β-diversity **(B)** in bulk and rhizosphere soils. NT, no-tillage; RT, ridge tillage; MP, moldboard plow; B, bulk soil; R, rhizosphere soil. The values are means of eight replicates, with different letters indicating significant differences at *p* < 0.05 (ANOVA).

Compared with NT, MP significantly decreased the diazotrophic α-diversity index in bulk soil, while RT showed little difference from NT (Figure 2A). In contrast, the three tillage practices showed no significant difference on α-diversity in rhizosphere soil (Figure 2A). Diazotrophic diversity had significantly negative correlations with the contents of soil TC, TN, C/N, TP, AP, AK, NH_4_^+^-N, and NO_3_^–^-N (Supplementary Figure S3). Additionally, the PCoA plot clearly showed that NT and RT formed a separate group from MP in bulk soil, while no obvious difference in diazotrophic community structures was observed among the tillage practices in rhizosphere soil (Figure 2B). The Adonis analysis revealed that larger differences were detected in NT *vs* MP (*F* = 6.728, *p* = 0.001) and RT *vs* MP (*F* = 6.643, *p* = 0.001) than in NT *vs* RT (*F* = 3.698, *p* = 0.001) as to bulk soil. The Mantel test revealed that all the soil properties examined significantly influenced the variations in diazotrophic community structure, except for TK (Supplementary Table S1).

### Analysis of Diazotrophs’ Network Structures

**FIGURE 3:**
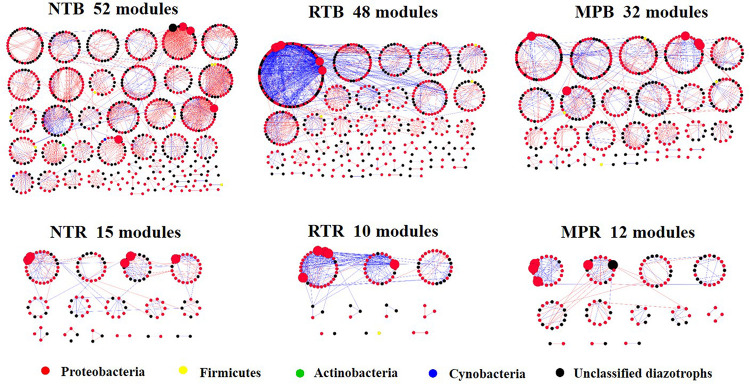
An overview of diazotrophs’ networks distributed by module under different tillage practices in bulk and rhizosphere soils. Each node represents an operational taxonomic unit, and the node colors represent major diazotrophic phyla. The blue line indicates a positive correlation between two individual nodes, whereas the red line indicates a negative correlation. Five nodes with the highest degree were magnified in each network, and the taxonomies of these nodes are detailed in Supplementary Table S3. NT, no tillage; RT, ridge tillage; MP, moldboard plow; B, bulk soil; R, rhizosphere soil.

The diazotrophic empirical networks revealed that the properties, in terms of average clustering coefficient, density, and modularity, were significantly different among the three tillage practices (*p* < 0.001) (Table 2). Specifically, MP network had fewer nodes and edges than those of NT and RT networks in bulk soils, while little difference in network complexity was detected in rhizosphere soils (Figure 3). The ratio of edge and node, average connectivity, clustering coefficient, and density of RT network were considerably higher than those of NT and MP for both bulk and rhizosphere soils (Table 2). The RT network also had more positive correlations among diazotrophic species and higher connectivity in terms of the distribution of degrees when compared with networks of NT and MP in both bulk and rhizosphere soils (Table 2 and Supplementary Figure S4). The node degree of the diazotrophs’ networks did not increase with the increase of the relative abundance of diazotrophic species, and the nodes with high degrees often had a low relative abundance (Supplementary Figure S5). The variations in soil properties induced by the three tillage practices had more influences on subnetwork in rhizosphere soil than on that in bulk soil (Supplementary Figure S6). Soil pH and AP were the most influential factors in the subnetwork of bulk soil, especially highly correlated with unclassified Proteobacteria and unclassified diazotrophs (Supplementary Table S2). In contrast, soil AK significantly affected the subnetwork in rhizosphere soil and had more correlations with species of *Bradyrhizobium* (Supplementary Figure S6). Furthermore, the nodes with the highest degree (top five) are displayed in Supplementary Table S3. Unlike networks of NT and MP, the nodes with top degree in the RT network were almost all positively correlated with other nodes in both bulk and rhizosphere soils (Supplementary Table S3). Specifically, OTU10838 (closely related to *Bradyrhizobium japonicum*), with the highest degree, was simultaneously found in the RT network of both bulk and rhizosphere soils and was positively correlated with other nodes.

**TABLE 2:** Major network properties of diazotrophs’ network under different tillage practices in bulk and rhizosphere soils.

**Network metrics**	**NTB**	**RTB**	**MPB**	**NTR**	**RTR**	**MPR**
Empirical network						
Similarity threshold (*S*_t_)	0.91	0.92	0.88	0.86	0.85	0.83
Number of nodes	792	579	501	149	97	129
Number of edges	1,980	1,536	907	246	302	204
Edge/node	2.50	2.65	1.81	1.65	3.11	1.58
*R*^2^ of power law	0.74	0.867	0.755	0.802	0.738	0.820
Number of positive edges	24.7%	68.2%	43.4%	62.6%	88.4%	71.1%
Number of negative edges	75.3%	31.8%	56.6%	37.4%	11.6%	28.9%
Average connectivity (avg*K*)	5.000	5.306	3.621	3.302	6.227	3.163
Average clustering coefficient (avgCC)	0.145^a^	0.163^a^	0.156^a^	0.229^b^	0.336^b^	0.195^b^
Density (*D*)	0.006^a^	0.009^a^	0.007^a^	0.022^b^	0.065^b^	0.025^b^
Modularity (*M*)	0.927^a^	0.673^a^	0.864^a^	0.755^b^	0.477^b^	0.775^b^
Random network^c^						
avgCC ± SD	0.010 ± 0.002	0.037 ± 0.004	0.008 ± 0.003	0.024 ± 0.011	0.126 ± 0.015	0.027 ± 0.012
*D* ± SD	0.004 ± 0.000	0.008 ± 0.000	0.006 ± 0.000	0.020 ± 0.000	0.061 ± 0.000	0.022 ± 0.000
*M* ± SD	0.438 ± 0.004	0.405 ± 0.005	0.553 ± 0.005	0.544 ± 0.011	0.306 ± 0.010	0.556 ± 0.012

*NT, no-tillage; RT, ridge tillage; MP, moldboard plow tillage; B, bulk soils; R, rhizosphere soils. ^*a*^Significant difference (*p* < 0.001) among tillage practices in bulk soil. ^*b*^Significant difference (*p* < 0.001) among tillage practices in rhizosphere soil. ^*c*^The random networks were generated by rewiring with identical numbers of the nodes and the links of the corresponding empirical network.*

## Discussion

### Differences in Soil Properties and Soybean Yields Among the Three Tillage Practices

No tillage has been commonly reported to increase soil nutrient storage with minimum mechanical soil disturbance compared with conventional tillage (Busari et al., 2015; Dang et al., 2015). However, in most studies, the collection of soil samples from near the surface of soils with crop residue coverage for NT or conventional tillage was conducted with no straw supply (Angers et al., 1997; Melero et al., 2011; Sun B. et al., 2016). Thus, the increased soil nutrients in the NT treatment appeared to be the result of the residue return effect rather than the reduced tillage (Mitchell et al., 2015). In this study, the crop residues were equally returned to the soil for all the three tillage practices, and tillage distributed the soil nutrients uniformly throughout the plow layer. The lower fertility at greater depths of NT resulted in lower contents of detected soil nutrients than in RT and MP (Table 1) because the soil samples of this study were collected from 0 to 20-cm depth rather than near the surface (0–5 cm) and were mixed thoroughly. Despite the reduction in tillage, consistent with the results of Mitchell et al. (2015), RT had similar soil nutrient contents to MP in both bulk and rhizosphere soils (Table 1). Aside from soil nutrients, MP resulted in higher soil pH than NT and RT in both bulk and rhizosphere soils in this research (Table 1). No consistent results have been found in previous studies on changes in soil pH in response to tillage practices (Cookson et al., 2008; Rahman et al., 2008), which might be the reason that tillage had no direct effect on soil pH but depended on soil type, climate conditions, and management factors. Additionally, Martínez et al. (2008) reported that conventional tillage promoted the ability of crop roots to acquire nutrients in deeper soil layers, therefore increasing root penetration and generating higher root mass. Although no significant change in soybean yield was observed among the three tillage practices (Supplementary Figure S1), insufficient access to nutrients might result in lower yields in NT than those in RT and MP, and the reduction in yield with NT remains a major concern (Singh et al., 2011; Pittelkow et al., 2015). In this context, RT is encouraged in terms of its benefits for increasing soil nutrients and improving crop yield compared to NT, and the minor soil disturbance in RT reduced the soil structure deterioration relative to MP.

### Comparative Analysis of Diazotrophic Composition and Structure of the Three Tillage Practices

Biological dinitrogen fixation performed by dinitrogen-fixing microorganisms is an initial process of the nitrogen cycle and the second largest nitrogen source after mineral fertilizers, which contributes up to 16% of the total global N supply (Ollivier et al., 2011). These microbes possess nitrogenase to exercise dinitrogen-fixing functions, in which the *nifH* gene encodes the subunit of nitrogenase and is highly conserved in prokaryotic microbes. Therefore, it becomes the marker gene of choice for diazotrophic communities in different ecosystems (Zehr et al., 2003; Collavino et al., 2014). In this study, Alphaproteobacteria and the genus *Bradyrhizobium* within this class dominated the diazotrophic communities (Figure 1), which had been reported to be the major diazotrophs in symbiosis with leguminous plants due to their strong persistence across diverse soil conditions (Pereira e Silva et al., 2013). Although higher soil nutrients were detected in both MP and RT than in NT, MP significantly changed the diazotrophic community composition, while RT had relatively similar diazotrophic communities compared to NT (Figures 1, 2). Compared to NT and RT, MP reduced the abundances of free-living diazotrophs, such as Betaproteobacteria and Cyanobacteria, while it promoted the growth of copiotrophic Alphaproteobacteria due to greater access to soil nutrients under MP (Figure 1) (Zuber and Villamil, 2016; Hutt et al., 2017; Che et al., 2018; Phoma et al., 2018). The differences in nutrient requirements of these diazotrophs were also confirmed by their correlations with soil properties (Supplementary Figure S2). Conversely, NT produced distinct soil stratification structures (Dang et al., 2015), which preserved a higher abundance of Actinobacteria (Figure 1A) that prefer a more stratified environment (Aislabie et al., 2006; Mathew et al., 2012).

Conventional tillage, with periodical soil inversion, leads to the homogenization of nutrient availability and also homogenizes the available microhabitats that have similar and specific microbes across the tillage fraction (Schmidt et al., 2019). Conversely, conservational tillage with no or little soil disturbance increases soil compartmentalization and generates a combination of surface enrichment (due to crop residue coverage) and subsoil nutrient depletion (Dang et al., 2015). These differences in soil stratification resulted in a lower diazotrophic diversity observed in MP than in NT and RT (Figure 2A). The negative influences of conventional tillage on microbial diversity had been reported in previous studies (Diosma et al., 2006; Wortman et al., 2013). The reduction of diazotrophic diversity in MP might be less able to cope with environmental pressures (Wang Y. et al., 2017), which was reflected in negative correlations between diazotrophic diversity and soil properties (Supplementary Figure S3). The changes in microbial community structure had been found to be determined by variations of soil properties in response to tillage practices (Lienhard et al., 2013), which was also detected in the results of the Mantel test of this research (Supplementary Table S1). In contrast to MP, RT did not significantly change the diazotrophic diversity and the community structures relative to NT despite the significant differences in soil properties between NT and RT in this study (Figure 2 and Table 1) or the microbial changes might recover to the status of NT after little disturbance of soils under RT (Wortmann et al., 2008). These results suggested that RT could preserve diazotrophic diversity as high as that of NT and might enhance the potential for microbial nutrient availability in the deeper subsoils, thereby promoting biological dinitrogen fixation (Dang et al., 2015). Noticeably, the changes in diazotrophic communities in response to tillage were mainly observed in bulk soils of this research, and little difference was detected in rhizosphere soils, which might be the stronger determinant of plant roots on diazotrophic communities by the nutrients, exudates, and mucilage released from roots than tillage practices (Philippot et al., 2013).

### Diazotrophs’ Network Properties and Interaction Correlations of the Three Tillage Practices

Microorganisms that coexist in complex interspecies interactions, either negative (e.g., competition) or positive (e.g., cooperation) (Faust et al., 2012), regulate or reflect the structure and the function of ecosystems to some great extent (Fuhrman, 2009). Our study showed that MP simplified the diazotrophs’ network structure relative to NT and RT (Figure 3). The complexity of networks often indicates a coordinated variability of microbial abundance which covary in response to interactions among species or environmental factors (Shi et al., 2016). Although the variations in soil properties showed similar trends in RT and MP, which were different from NT (Table 1), RT resulted in a more complex diazotrophs’ network than MP in bulk soil. This was likely attributed to the diazotrophic species interactions rather than the soil properties controlling the variation of diazotrophs’ networks. Nevertheless, we found that the variations in soil properties induced by the three tillage practices had more effects on diazotrophic interactions in rhizosphere soil than in bulk soil (Supplementary Figure S6). This phenomenon might be expected to spread disturbances quickly through the rhizosphere network once the environment is disrupted and has difficulty in recovering (Fan et al., 2018). In addition, the higher connectivity, clustering coefficient, and density in RT network, than the networks of MP and NT, indicated the much tighter interactions, such that diazotrophic species intensely and efficiently affected each other under RT (He et al., 2017). These network properties could reflect extensively cooperative relationships among members (Banerjee et al., 2018), which was also observed in RT network with more positive correlations than the networks of NT and MP (Table 2). The cooperative characteristic of the diazotrophs’ network in RT improved the fluxes of energy, material, and information with cooperative interactions among diazotrophic assemblages (Rasmann and Turlings, 2016; Zheng et al., 2018). In addition, the extensive cooperation in RT was also reflected in individual nodes, especially in the node with the highest degree (Supplementary Table S3). For example, *B. japonicum* had more interactions detected in both bulk and rhizosphere soils than any other diazotrophic species (Supplementary Table S3). This diazotrophic species has the ability to adapt to various environments with multiple survival strategies and can improve soybean nodulation and enhance soybean plant growth by co-inoculation of *Bacillus* strains (Bai et al., 2003). Nevertheless, due to the limited database, the unclassified diazotrophic species should be further examined to better understand the influences of different tillage practices on diazotrophic communities and networks.

## Conclusion

In the current study, RT and MP significantly increased the contents of most nutrients in the plow layer (0–20 cm) compared to NT. No obvious effect of RT on diazotrophic taxa, community diversity, and structure relative to NT was observed, but MP induced lower diazotrophic diversity and obviously changed the diazotrophic communities in comparison with NT and RT. MP promoted the growth of copiotrophic diazotrophs but was not favorable for the growth of free-living diazotrophs compared with NT and RT. In addition, MP simplified the diazotrophs’ network structures, which was consistent with the decrease in diazotrophic diversity when compared with NT and RT in bulk soils. In contrast, RT network induced tighter correlations among diazotrophic species with more cooperative interactions than the networks of NT and MP. The extensive cooperation in the RT network was mainly associated with the individual node of *B. japonicum* detected in both bulk and rhizosphere soils. This research revealed that RT with minor soil disturbance might be a suitable soil tillage practice for improving soil nutrients, stabilizing diazotrophic communities, and increasing cooperative interactions among diazotrophic species relative to NT and MP.

## Data Availability Statement

Publicly available datasets were analyzed in this study. These data can be found in the NCBI under accession number SRP226893.

## Author Contributions

JL and AL conceptualized the study. JL, GW, AL, and XH designed the experiments. XH, QY, and ZL performed the experiments. XH, QY, and ZY interpreted the results. JL, AL, and XH wrote the manuscript. GW and JL revised the manuscript. All the authors approved the final manuscript as submitted.

## Conflict of Interest

The authors declare that the research was conducted in the absence of any commercial or financial relationships that could be construed as a potential conflict of interest.

## Footnotes

1 http://qiime.sourceforge.net/

2 http://fungene.cme.msu.edu/

3 http://fungene.cme.msu.edu/FunGene

4 http://ieg4.rccc.ou.edu/mena

## Supplementary Material

FIGURE S1 Effects of different tillage practices on soybean yields in the sampling year. NT, no tillage; RT, ridge tillage; MP, moldboard plow. The values are means of eight replicates, with different letters indicating significant differences at *p* < 0.05 (ANOVA).

FIGURE S2 Correlations between diazotrophic phyla, genera, and soil properties. Significant correlations based on Pearson’s correlation coefficients (^∗^*p* < 0.05; ^∗∗^*p* < 0.01) and *r* values are shown in bold.

FIGURE S3 Correlations between α-diversity index and soil properties. Significant correlations calculated by Pearson’s correlation coefficients (^∗^*p* < 0.05; ^∗∗^*p* < 0.01).

FIGURE S4 Distribution of node degree in each diazotrophs’ network. In bulk soil, the number of degrees was limited to a maximum of 22, 56, and 17 in the NT, RT, and MP, respectively; in rhizosphere soil, the number of degrees was limited to a maximum of 13, 21, and 13 in the NT, RT, and MP, respectively. NT, no tillage; RT, ridge tillage; MP, moldboard plow; B, bulk soil; R, rhizosphere soil.

FIGURE S5 Correlations between relative abundances of diazotrophic nodes and their degrees in each diazotrophs’ network. NT, no tillage; RT, ridge tillage; MP, moldboard plow; B, bulk soil; R, rhizosphere soil.

FIGURE S6 Subnetwork showing the influences of the variation in soil properties under different tillage practices on diazotrophs’ networks in bulk and rhizosphere soils. Each node represents an operational taxonomic unit, and the node colors represent diazotrophic genera. Nodes with direct links to soil properties are magnified in each subnetwork. The blue line indicates a positive correlation between two individual nodes, whereas the red line indicates a negative correlation.

TABLE S1 Mantel test to determine the correlation between diazotrophic community structure and soil properties in soils under different tillage practices.

TABLE S2 Correlations between diazotrophic networks and soil properties in the bulk and rhizosphere soils.

TABLE S3 Information of nodes with highest degree (top 5) in each diazotrophic network.
