# Supplementary material for: Ridge Tillage Improves Soil Properties, Sustains Diazotrophic Communities, and Enhances Extensively Cooperative Interactions Among Diazotrophs in a Clay Loam Soil
Source: Front Microbiol. 2020 Jun 30;11:1333. doi: 10.3389/fmicb.2020.01333 (PMC7344147; doi:10.3389/fmicb.2020.01333)
Supplement: FIGURE S1 — Effects of different tillage practices on soybean yields in the sampling year. NT, no tillage; RT, ridge tillage; MP, moldboard plow. The values are means of eight replicates, with different letters indicating significant differences at p < 0.05 (ANOVA). [file Presentation_1.PPTX]

## Slide 1
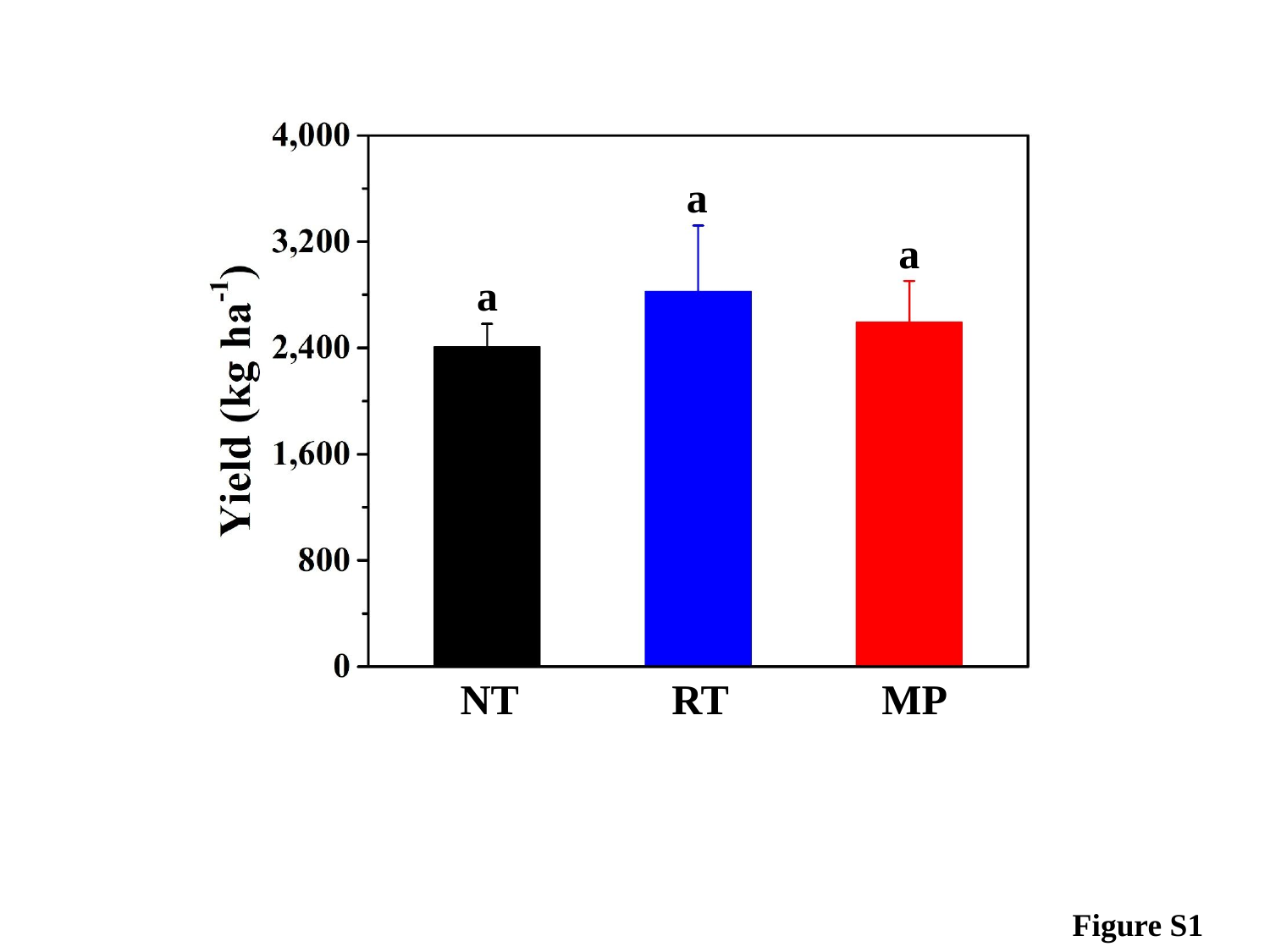

a
a
a
NT
RT
MP
Figure S1

## Slide 2
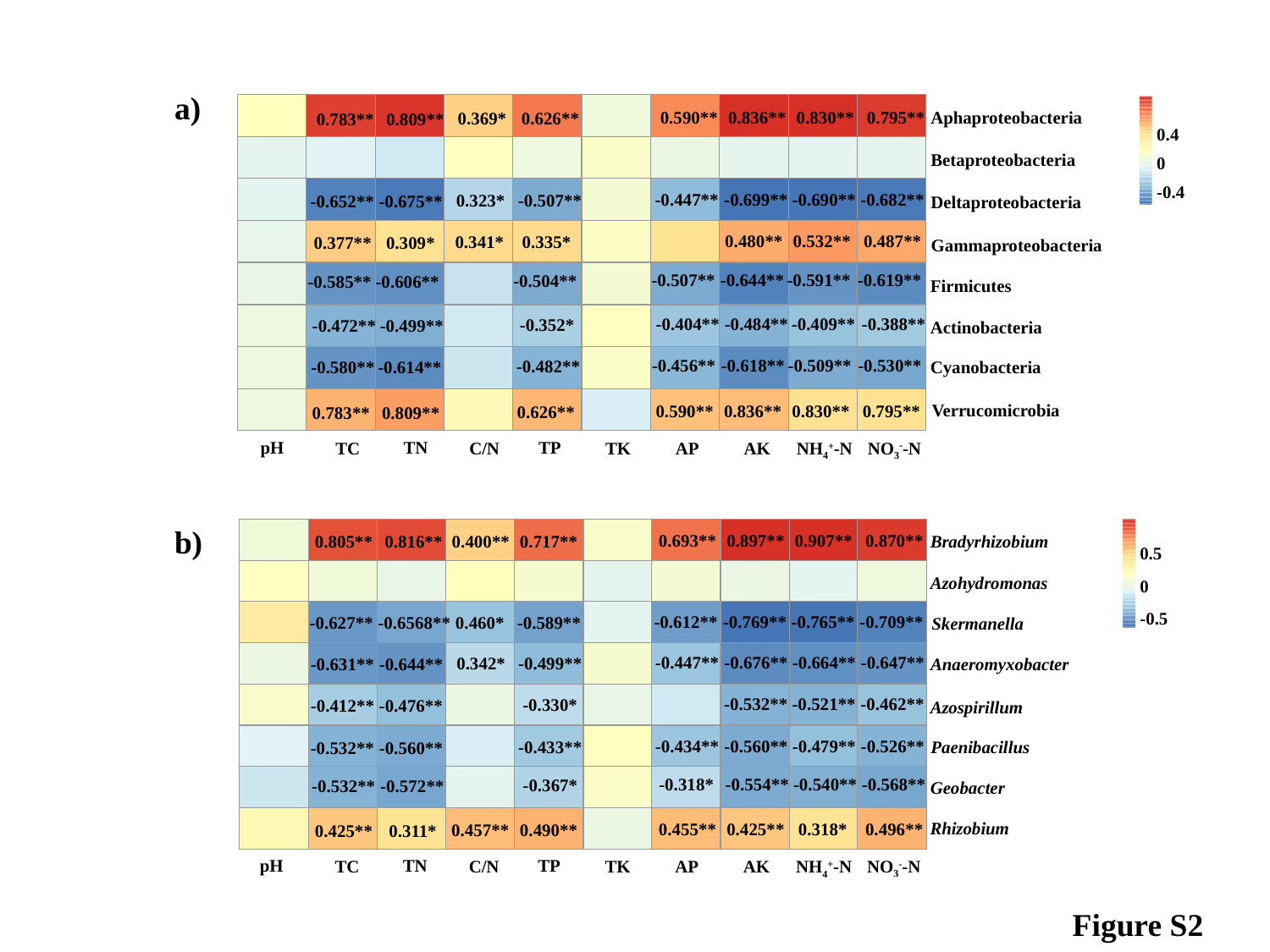

a)
0.590**
0.836**
0.830**
0.795**
0.369*
0.626**
0.783**
0.809**
-0.447**
-0.699**
-0.690**
-0.682**
0.323*
-0.507**
-0.652**
-0.675**
0.480**
0.532**
0.487**
0.341*
0.335*
0.377**
0.309*
-0.507**
-0.644**
-0.591**
-0.619**
-0.504**
-0.585**
-0.606**
-0.404**
-0.484**
-0.409**
-0.388**
-0.352*
-0.472**
-0.499**
-0.456**
-0.509**
-0.530**
-0.482**
-0.580**
-0.614**
0.590**
0.836**
0.830**
0.795**
0.626**
0.783**
0.809**
0.4
0
-0.4
Aphaproteobacteria
Betaproteobacteria
Deltaproteobacteria
Gammaproteobacteria
Firmicutes
Actinobacteria
-0.618**
Cyanobacteria
Verrucomicrobia
TP
pH
TN
TC
C/N
NO3--N
TK
AP
AK
NH4+-N
Bradyrhizobium
Azohydromonas
Skermanella
Anaeromyxobacter
Azospirillum
Paenibacillus
Geobacter
Rhizobium
TP
pH
TN
TC
C/N
NO3--N
TK
AP
AK
NH4+-N
0.693**
0.897**
0.907**
0.870**
0.400**
0.717**
0.805**
0.816**
-0.612**
-0.769**
-0.765**
-0.709**
0.460*
-0.589**
-0.627**
-0.6568**
-0.447**
-0.676**
-0.664**
-0.647**
0.342*
-0.499**
-0.631**
-0.644**
-0.532**
-0.521**
-0.462**
-0.330*
-0.412**
-0.476**
-0.434**
-0.560**
-0.479**
-0.526**
-0.433**
-0.532**
-0.560**
-0.318*
-0.554**
-0.540**
-0.568**
-0.367*
-0.532**
-0.572**
0.455**
0.425**
0.318*
0.496**
0.457**
0.490**
0.425**
0.311*
b)
0.5
0
-0.5
Figure S2

## Slide 3
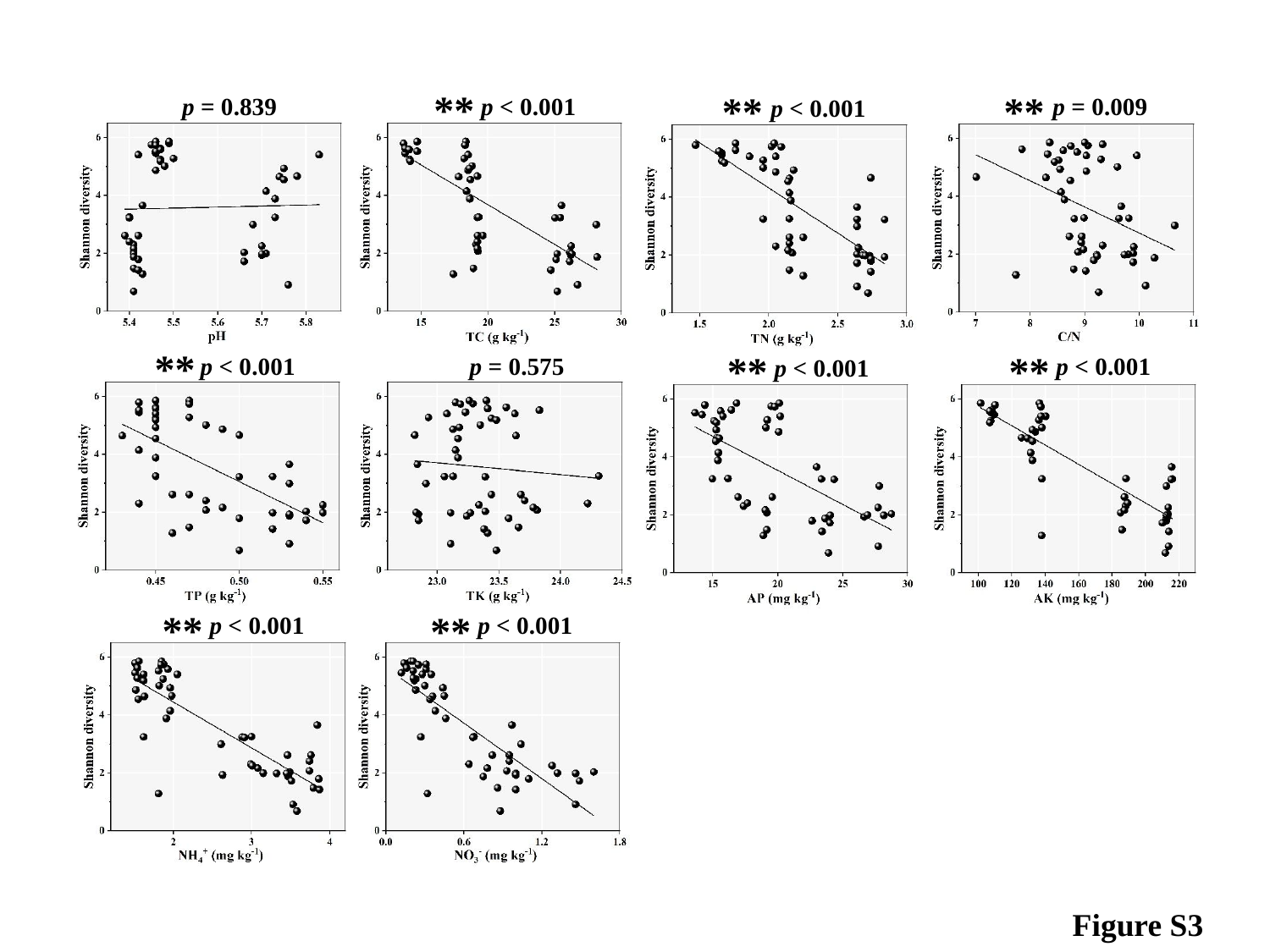

**
p < 0.001
**
p < 0.001
**
p = 0.009
p = 0.839
**
p < 0.001
**
p < 0.001
**
p < 0.001
p = 0.575
**
p < 0.001
**
p < 0.001
Figure S3

## Slide 4
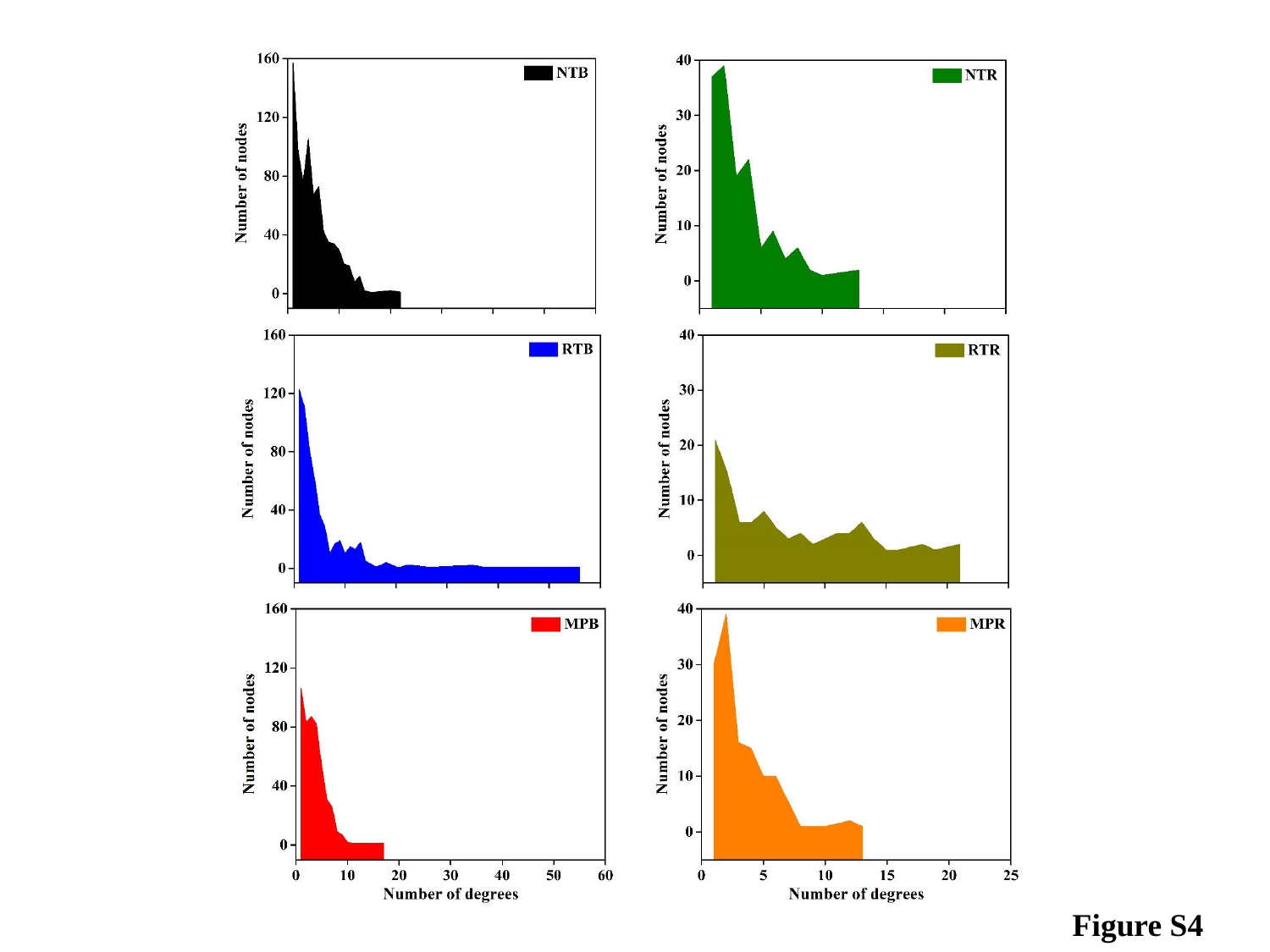

Figure S4

## Slide 5
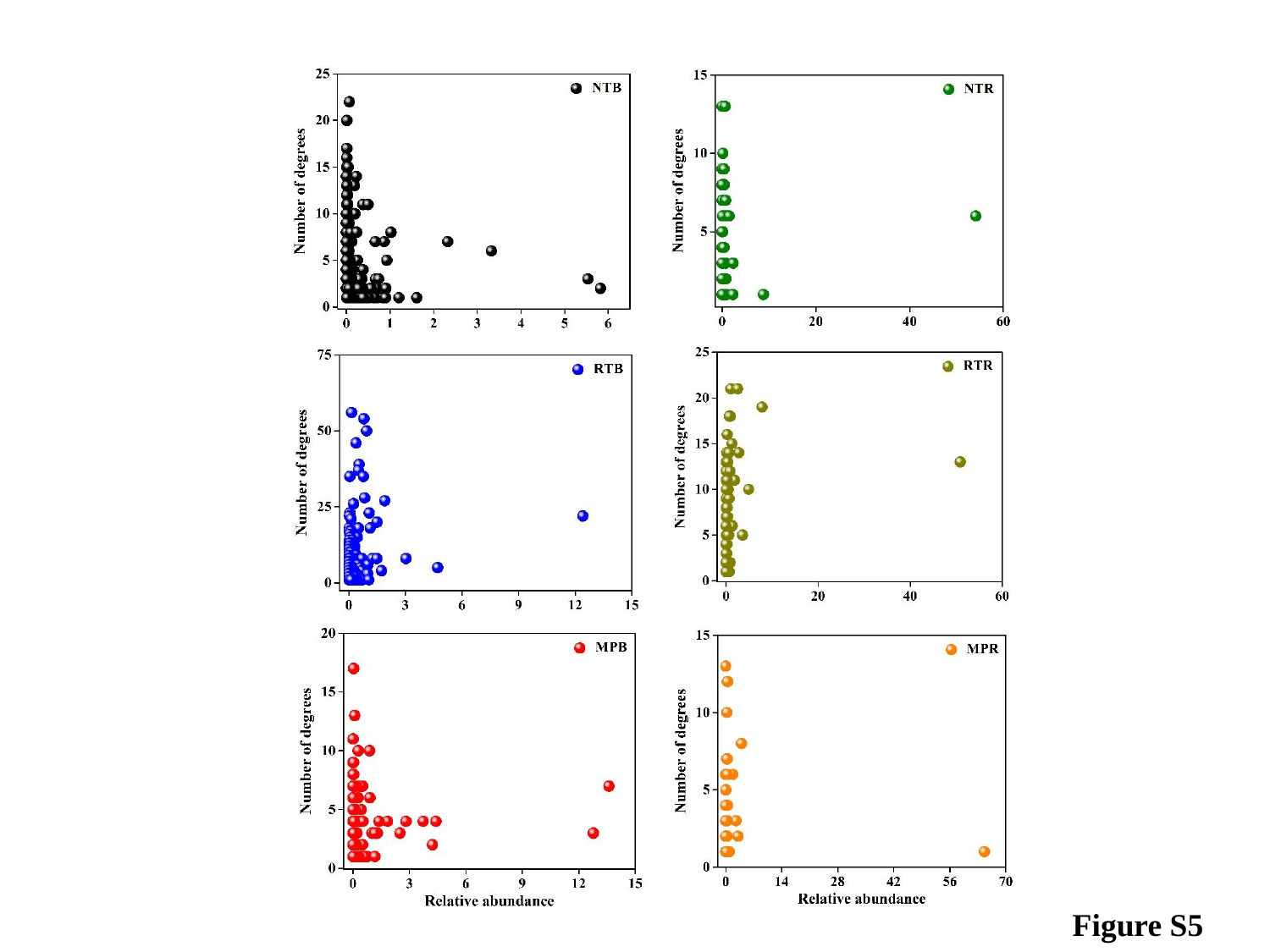

Figure S5

## Slide 6
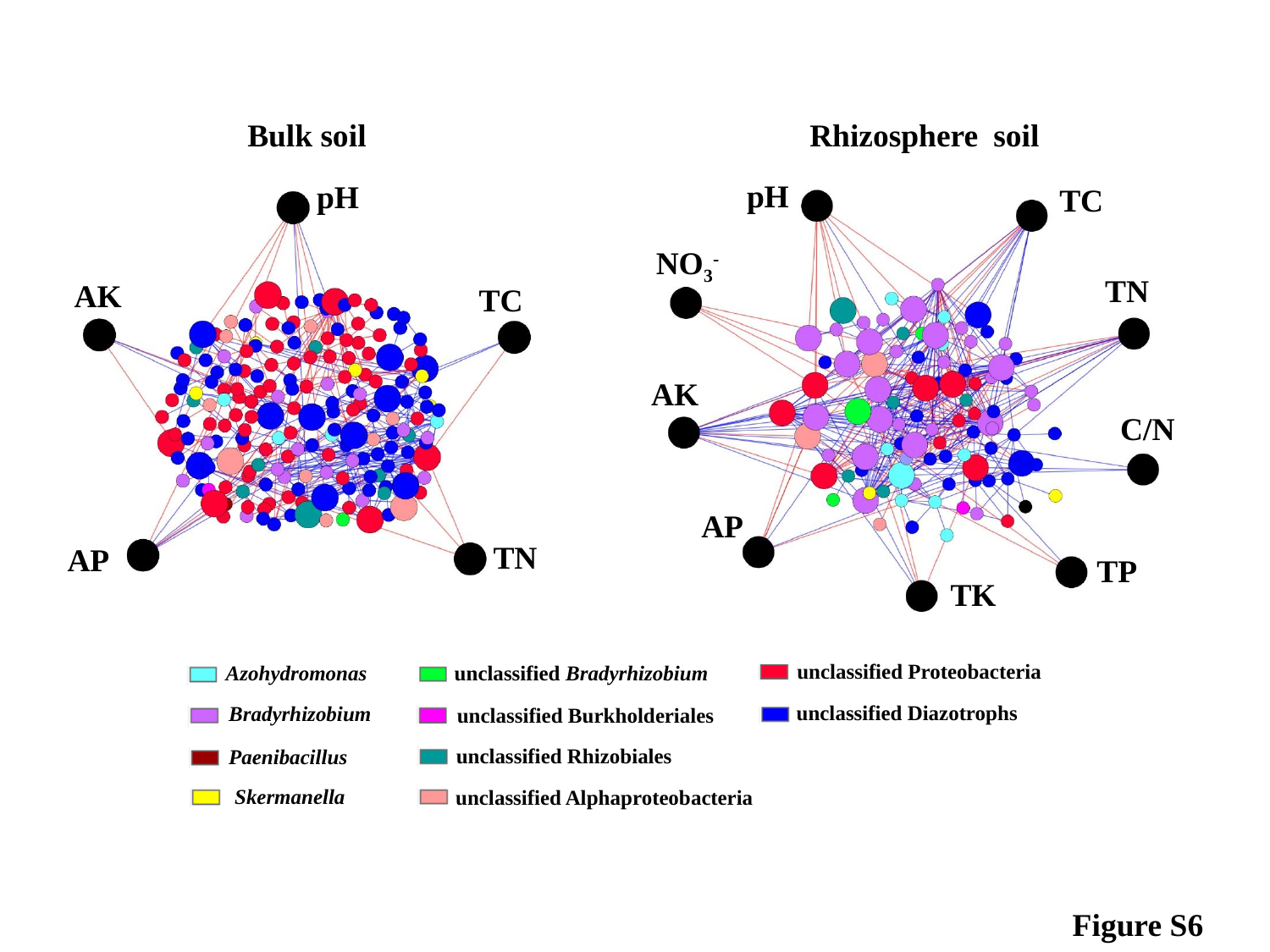

Bulk soil
pH
AK
TC
TN
AP
Rhizosphere soil
pH
TC
NO3-
TN
AK
C/N
AP
TP
TK
unclassified Proteobacteria
Azohydromonas
unclassified Bradyrhizobium
unclassified Diazotrophs
Bradyrhizobium
unclassified Burkholderiales
unclassified Rhizobiales
Paenibacillus
Skermanella
unclassified Alphaproteobacteria
Figure S6
