# Supplementary material for: Ridge Tillage Improves Soil Properties, Sustains Diazotrophic Communities, and Enhances Extensively Cooperative Interactions Among Diazotrophs in a Clay Loam Soil
Source: Front Microbiol. 2020 Jun 30;11:1333. doi: 10.3389/fmicb.2020.01333 (PMC7344147; doi:10.3389/fmicb.2020.01333)
Supplement: TABLE S1 — Mantel test to determine the correlation between diazotrophic community structure and soil properties in soils under different tillage practices. [file Table_1.DOCX]

**Table S1** Mantel test to determine the correlation between diazotrophic community structure and soil properties in soils under different tillage practices.

| Soil properties |  | *r* | *p*-value |
| --- | --- | --- | --- |
| pH |  | **0.097** | 0.017 |
| TC |  | **0.326** | 0.001 |
| TN |  | **0.348** | 0.001 |
| C/N |  | **0.114** | 0.014 |
| TP |  | **0.258** | 0.001 |
| TK |  | 0.034 | 0.227 |
| AP |  | **0.288** | 0.001 |
| AK |  | **0.693** | 0.001 |
| NH_4_^+^-N |  | **0.652** | 0.001 |
| NO_3_^-^-N |  | **0.495** | 0.001 |

*Significant correlation (p < 0.05) were shown in bold.*

**Table S2** Correlations between diazotrophic networks and soil properties in the bulk and rhizosphere soils.

|  | Correlation | pH | TC | TN | C/N | TP | TK | AP | AK | NH_4_^+^-N | NO_3_^-^-N |
| --- | --- | --- | --- | --- | --- | --- | --- | --- | --- | --- | --- |
| Bulk soil | Positive | 2 | 2 | 0 | 0 | 0 | 0 | 4 | 2 | 0 | 0 |
|  | Negative | 5 | 1 | 3 | 0 | 0 | 0 | 3 | 2 | 0 | 0 |
| Rhizosphere soil | Positive | 1 | 9 | 9 | 3 | 1 | 2 | 2 | 13 | 0 | 0 |
|  | Negative | 8 | 4 | 4 | 0 | 2 | 3 | 4 | 3 | 0 | 4 |

**Table S3** Information of nodes with highest degree (top 5) in each diazotrophic network.

|  | Treatments | Nodes | Positive degree | Negative degree | Taxa |
| --- | --- | --- | --- | --- | --- |
| Bulk soil | NTB | OTU11085 | 0 | 22 | Unclassified diazotroph |
|  |  | OTU6954 | 0 | 20 | Proteobacteria |
|  |  | OTU7106 | 0 | 20 | Proteobacteria |
|  |  | OTU2192 | 2 | 15 | Proteobacteria |
|  |  | OTU10124 | 1 | 15 | Proteobacteria |
|  | RTB | OTU6243 | 56 | 0 | Proteobacteria |
|  |  | OTU10838 | 54 | 0 | *Bradyrhizobium* |
|  |  | OTU11071 | 50 | 0 | *Azohydromonas* |
|  |  | OTU5027 | 46 | 0 | *Bradyrhizobium* |
|  |  | OTU8787 | 39 | 0 | Proteobacteria |
|  | MPB | OTU3824 | 15 | 2 | Proteobacteria |
|  |  | OTU4887 | 13 | 0 | *Bradyrhizobium* |
|  |  | OTU2316 | 1 | 10 | Proteobacteria |
|  |  | OTU5007 | 10 | 0 | *Skermanella aerolata* |
|  |  | OTU5703 | 10 | 0 | *Skermanella aerolata* |
| Rhizosphere soil | NTR | OTU2425 | 11 | 2 | Proteobacteria |
|  |  | OTU10719 | 7 | 6 | *Bradyrhizobium* |
|  |  | OTU5634 | 1 | 9 | *Azohydromonas* |
|  |  | OTU11232 | 8 | 1 | *Bradyrhizobium* |
|  |  | OTU10812 | 7 | 2 | Bradyrhizobiaceae |
|  | RTR | OTU10838 | 21 | 0 | *Bradyrhizobium* |
|  |  | OTU11090 | 20 | 1 | Bradyrhizobiaceae |
|  |  | OTU6470 | 18 | 1 | *Bradyrhizobium* |
|  |  | OTU4129 | 17 | 1 | Bradyrhizobiaceae |
|  |  | OTU11011 | 17 | 1 | *Azohydromonas* |
|  | MPR | OTU11194 | 7 | 6 | *Rhizobium* |
|  |  | OTU11090 | 12 | 0 | *Bradyrhizobium* |
|  |  | OTU10838 | 12 | 0 | Bradyrhizobiaceae |
|  |  | OTU4983 | 8 | 2 | *Azohydromonas* |
|  |  | OTU6470 | 8 | 0 | Bradyrhizobiaceae |

*NT, no-tillage; RT, ridge tillage; MP, moldboard plow tillage; B, bulk soils; R, rhizosphere soils.*
